# Acute exercise in mice transiently remodels the hepatic lipidome in an intensity-dependent manner

**DOI:** 10.1186/s12944-020-01395-4

**Published:** 2020-10-08

**Authors:** Gregory C. Henderson, Valeria Martinez Tenorio, Marc A. Tuazon

**Affiliations:** 1 Department of Nutrition Science, Purdue University, 700 West State Street, West Lafayette, IN 47907 USA; 2 Department of Nutritional Sciences, Rutgers University, New Brunswick, NJ 08901 USA

**Keywords:** Lipidomics, High-intensity interval training, Postexercise recovery, Post-exercise, Intrahepatocellular lipid, Triglyceride

## Abstract

**Background:**

The content of triacylglycerol (TAG) in the liver is known to rapidly increase after a single bout of exercise followed by recovery to sedentary levels. The response of other hepatic lipids, and acyl chain composition of lipid classes, would provide a deeper understanding of the response of hepatic lipid metabolism to acute exercise.

**Methods:**

Female mice performed a single bout of continuous exercise (CE), high-intensity interval exercise (HIIE), or no exercise (CON). The total content of various lipids in the liver, and fatty acids within lipid classes, were measured in tissues collected 3 h after exercise (Day 1) and the day following exercise (Day 2).

**Results:**

The total concentration of TAG rose on Day 1 after exercise (*P* < 0.05), with a greater elevation in HIIE than CE (*P* < 0.05), followed by a decline toward CON levels on Day 2. The total concentration of other measured lipid classes was not significantly altered by exercise. However, n-6 polyunsaturated fatty acid relative abundance in diacylglycerol (DAG) was increased by HIIE (*P* < 0.05). In CON liver, TAG content was positively correlated with DAG and phosphatidylethanolamine (*P* < 0.05), while these statistical associations were disrupted in exercised mice on Day 1.

**Conclusions:**

The response of lipid metabolism to exercise involves the coordination of metabolism between various tissues, and the lipid metabolism response to acute exercise places a metabolic burden upon the liver. The present findings describe how the liver copes with this metabolic challenge. The flexibility of the TAG pool size in the liver, and other remodeling of the hepatic lipidome, may be fundamental components of the physiological response to intense exercise.

## Background

The lipid that accumulates in the body during positive energy balance is primarily as triacylglycerol (TAG) in adipose tissue. However, TAG and other lipids can accumulate elsewhere as well at lower concentrations, and this ectopic lipid deposition is common during weight gain and other states of metabolic dysfunction in laboratory rodents and human subjects [1–6]. This lipid accumulation has serious ramifications for metabolism; excess TAG accumulation in liver and muscle typically presents alongside elevated levels of lipotoxic intermediates such as diacylglycerol (DAG) [1–5]. DAG and even perhaps other lipotoxic intermediates in these insulin-responsive tissues can lead to the development of insulin resistance [2, 7, 8]. Structural membrane lipids, such as phospholipid, are less likely to be responsive to lifestyle factors that drive weight gain or weight loss. However, phospholipids do coat intracellular lipid droplets and therefore could play a role in TAG accumulation within various tissues. The responses of tissue TAG concentrations to lifestyle factors that alter lipid metabolism have been studied in depth in both laboratory rodents and human subjects, such as the net utilization of intramuscular TAG with acute exercise [9–13] and the transient accumulation of hepatic TAG in response to an acute exercise bout [11, 13–17]. In contrast to acute effects of exercise, chronic exercise training of human subjects appears to elevate intramuscular TAG [6, 18] and can potentially reduce hepatic TAG to a modest extent in certain instances [6, 19]. However, it must be noted that chronically training individuals could potentially experience acute effects of each exercise bout. Thus, in individuals undertaking an exercise training program, meaningful fluctuations in the content of these lipid depots are expected to occur during the course of each day, as a result of their exercise participation. The liver’s ability to transiently expand its TAG content during and after exercise deserves particular attention, as this is a more recently appreciated component of the exercise response. In order to develop a better understanding of this phenomenon, a greater level of knowledge is needed regarding the complexity of the lipid profile in the liver and its acute response to exercise bouts.

While lipid content in the liver and other peripheral tissue is of great importance for metabolic health, the largest lipid-enriched fuel depot in the body is adipose tissue. TAG mobilization through lipolysis in adipose tissue is enhanced during exercise and post-exercise recovery [20–22]. This lipolysis response, driven by catecholamines and other endocrine stress responses, likely evolved to match the mobilization of free fatty acids (FFA) to the fuel needs of exercised skeletal muscle. While expression level of FFA transport proteins is expected to play some role in tissue FFA uptake [23], FFA concentration gradients and effects of mass action would still play critical roles in the control of FFA uptake into tissues. Plasma FFA concentration is elevated during exercise to drive FFA from adipose tissue to working skeletal muscle, yet the increased circulating FFA will also drive this lipid to other organs by mass action. The ability to cope with enhanced FFA supply to non-muscle tissues during exercise is metabolically critical but has not been addressed extensively in metabolic research. Mobilization of FFA during exercise appears to be approximately matched to skeletal muscle’s needs, but to achieve this appropriate supply of FFA to skeletal muscle, the FFA supply to other tissues may exceed their needs. This could explain why hepatic TAG acutely accumulates during and after exercise in laboratory animals [9, 17, 24], followed by a spontaneous return of hepatic TAG back to near sedentary levels by the day after exercise as recovery from the stressor takes place [17]. Furthermore, blocking lipolysis with nicotinic acid administration prevents this exercise-induced hepatic lipid accumulation in rats [24], further supporting the notion that exercise-induced lipolysis leads to the accumulation of lipid outside of exercised muscle. Other controllers of hepatic TAG content would be fatty acid oxidation and synthesis, as well as lipoprotein secretion. However, FFA uptake appears to be the most likely primary explanation for exercise-induced liver TAG accumulation. In human subjects studied by magnetic resonance spectroscopy (MRS) before and after exercise [11, 13–16], the accumulation of hepatic lipid has been observed, in agreement with the work on both rats and mice [9, 17, 24]. Providing the appropriate amount of FFA to contracting muscle through accentuated lipolysis in adipose tissue even leads to an apparent excess of FFA supply to the non-exercised resting muscle, as resting muscle during exercise accumulates TAG [12]. Clearly there is a need to develop a more elaborate understanding of the metabolic stress that is placed upon various organs during and after exercise, including those tissues that must cope with a net accumulation of TAG.

While clinically it is of interest to devise approaches to chronically reduce the level of TAG accumulation in the liver, it should not be ignored that substantial day-to-day and hour-to-hour fluctuations in hepatic TAG and other lipids may also be very important for support of metabolic integration and overall health. The ability to rapidly increase lipid storage at ectopic lipid deposition sites, when metabolically appropriate, is a physiological parameter that deserves increased attention; the term “lipogenic flexibility” is proposed for this emerging phenomenon in stress physiology. While lipogenic flexibility is depicted primarily by transient changes in tissue TAG, broadly testing the lipid profile could provide a deeper understanding of the hepatic response to acute exercise. As it has become clear that hepatic TAG can increase substantially as a transient response after exercise, the objective of the study reported here was to discover the relationship between this TAG response and that of other lipid classes in the liver. In the present report, results from testing a broad range of lipid classes and their acyl chain composition are presented; these investigations were carried out to further characterize the response of hepatic lipids to an acute exercise bout in mice. Endurance exercise can be performed as continuous exercise (CE) or as high-intensity interval exercise (HIIE). As discussed previously, both exercise types can be employed in patients, and the metabolic impacts of HIIE might be expected to be more potent than CE [17]. Therefore, in the present pre-clinical study of exercise responses, both CE and HIIE were tested. It was hypothesized that a single bout of exercise would impact the hepatic lipidome, with a more potent impact of HIIE than CE.

## Methods

### Animals

This protocol was approved by the Rutgers University Institutional Animal Care and Use Committee. Initial results from the study were published previously [17]. Following initial study execution, lipidomics analysis was performed on liver samples from the female mice in the study, and those results are presented here and have not been published previously. C57BL/6 J mice were purchased from the Jackson Laboratory (Bar Harbor, ME, USA) and housed in the animal facility at Rutgers University. The mice were maintained on a 12-h light/dark photoperiod with all mice allowed ad libitum access to food and water. All mice consumed the Labdiet 5 K52 diet (Purina Mills, Richmond, IN, USA); the fatty acid profile of this diet has been reported previously [25]. No chronic exercise training was imposed; the mice were sedentary for their entire lives, with the exception of the single acute exercise bout described below for mice assigned to CE or HIIE groups. Mice were acclimated to the facility for at least 5 days prior to exercise.

### Exercise protocols

Mice were exercised between the ages of 14–16 weeks on a treadmill (Exer-3/6, Columbus Instruments, Columbus, OH, USA) with a shock grid set at a low intensity (on a scale of 0–10, set at 1). On the day before exercise, all mice were acclimated to the treadmill for 5 min at a speed of 5 m/min with no incline (0°). Subsequently, mice were then assigned to sedentary control (CON), CE, or HIIE groups. Food was withdrawn at 7:00 AM on the day of exercise. For mice assigned to CE or HIIE, the exercise session began between 11:45 AM and 12:30 PM. CON was a time-of-day matched condition in which mice remained in their cages with water bottles withdrawn for the amount of time that CE and HIIE mice would spend away from their cages during the exercise session (approximately 35 min). For HIIE, following a 5 min warmup at a slow walking speed (5 m/min), mice ran for 30 s intervals with 60 s walking rest periods (5 m/min) interspersed between intervals. The exercise session included 20 running intervals, the first at 15 m/min, next at 20 m/min, then at 25 m/min, followed by all remaining sprint intervals at a final speed of 30 m/min. Acceleration up to 15, 20, 25 and 30 m/min was performed within a 5 s ramping duration and deceleration back to 5 m/min within a 2 s duration. Both the warmup and the exercise session were performed at an incline of 25°. CE consisted of an incline-matched, duration-matched, and distance-matched continuous running session (13.8 m/min for 30 min) following the same 5 min warmup phase. These HIIE and CE protocols have been confirmed previously to also be matched for energy expenditure in mice [26]. To avoid repeated shocks and to maintain running speed, mice were gently prodded by hand when they closely approached the shock grid.

### Tissue collection

On one set of mice, euthanasia followed by liver tissue collection was performed on the day of exercise (Day 1) while on a separate set of mice the euthanasia and tissue collection were performed on the day following exercise (Day 2). For the mice assigned to tissue collection on Day 1, they remained fasted for 8 h until euthanasia at 3:00 PM. For mice assigned to tissue collection on Day 2, on the day of exercise they were returned to their cages and given food at 7:00 PM for overnight free food access; the following morning, food was again withdrawn at 7:00 AM and then euthanasia was performed at 3:00 PM following an 8-h fast. Once collected, liver tissues were immediately frozen in liquid nitrogen then stored at − 80 °C until analysis. Liver tissue from a total of 36 female mice was collected analyzed (*n* = 6 per condition on Day 1, and *n =* 6 per condition on Day 2).

### Lipid analysis

Liver samples from 6 mice per group were sent to Metabolon (Lipomics Inc., Sacramento, CA, USA) for lipid profile analysis. Lipids were extracted from liver tissue, fractionated into specific lipid classes, followed by derivatization of the acyl chains to fatty acid methyl esters (FAME). The derivatized fatty acids from each lipid class were then analyzed by gas chromatography by a previously published method [27]. The total content of each lipid class was calculated (μmol per gram of liver wet weight) as well as the fatty acid composition (mol %) for each of the following lipid classes: TAG, DAG, cholesterol ester (ChE), cardiolipin (CL), lysophosphatidylcholine (LyPC), phosphatidylcholine (PC), phosphatidylethanolamine (PE), phosphatidylserine (PS), and sphingomyelin (SM).

### Statistical analysis

Data are presented as means ± standard error. The total content of each lipid class in the liver was analyzed by 2-way (time-by-trial) analysis of variance (ANOVA). Both factors in the ANOVA were between-group factors. Within a lipid class, the relative abundance of saturated fatty acids (SFA), monounsaturated fatty acids (MUFA), and polyunsaturated fatty acids (PUFA) were tested with the same ANOVA structure. The time level in ANOVAs had 2 factors (Day 1 vs Day 2) and the trial level had 3 factors (CON, CE, HIIE). Main effect of trial and trial-by-time interactions were tested. ANOVA was followed by Fisher’s Least Significant Difference (LSD) post hoc test. The Pearson-product moment correlation coefficient (r) was used to quantify the strength of relationships between the total content of hepatic TAG and other lipid classes, and the statistical significance of r was described by the corresponding *P*-value. Correlation coefficients were converted to *R*^*2*^ for reporting in Results. Correlations were performed for sedentary controls alone (CON data from Day 1 and 2 pooled); if there was a significant correlation in CON mice, next the potential correlation in post-exercise Day 1 samples (CE and HIIE groups pooled) was tested to ascertain if the association was maintained during the expansion of the hepatic TAG pool after exercise. Correlation in post-exercise Day 2 samples (CE and HIIE groups pooled) was also tested to ascertain if the association was maintained a day after exercise. For ANOVA and linear regression, the statistical analyses were performed with JMP version 8 (SAS Institute Inc., Cary, NC, USA) and *P* < 0.05 was considered statistically significant. In linear regression, while *P* < 0.05 was considered to be statistically significant, in the results interpretation a *P*-value of 0.05–0.10 was considered to suggest a potential trend toward significance. Principal component analysis (PCA) was performed on the relative fatty acid composition of lipid classes using Metabo-Analyst 4.0 software. Fatty acids with a relative abundance greater than or equal to 0.1% and a variable importance in the projection (VIP) score greater than 1 were included in the PCA.

## Results

Body composition of the mice (10.4 ± 0.4% body fat) was reported previously [17]. Hepatic TAG content from the same mice studied here, measured by an enzyme-linked colorimetric assay, was reported previously [17]. For the lipidomics analysis that is presented in this report, a new piece of tissue was cut from each of these original liver samples, and underwent lipid extraction and then analysis by chromatographic techniques that are distinct from the previous colorimetric approach. Previously, the only lipid measured was TAG, while here results from a broad lipid profile are reported. These independently performed analyses of TAG from the same mouse livers provide an opportunity for method comparison. The present results for group differences in TAG (Table 1) are in close agreement with the previously reported findings [17], despite the fact that the analyses were performed in different laboratories, by different methods, and by different analysts. To further explore the method comparison, linear regression was performed to compare results for each liver sample from the two methods (total of 36 samples). The correlation was significant with a very large correlation (*P* < 0.0001, *R*^*2*^ = 0.86, slope = 0.99).

**Table 1:** Total content of lipid classes in the liver

		TAG^^&^	DAG	ChE	CL	LyPC	PC	PE	PS	SM
*Day 1*	CON	16.7 ± 2.1	3.9 ± 0.4	4.0 ± 0.3	3.2 ± 0.3	1.6 ± 0.1	27.2 ± 3.0	15.0 ± 0.3	3.3 ± 0.3	1.5 ± 0.04
	CE	24.1 ± 2.2*	4.7 ± 0.4	4.7 ± 0.4	3.2 ± 0.4	1.5 ± 0.1	21.9 ± 2.0	14.4 ± 0.3	3.2 ± 0.2	1.4 ± 0.1
	HIIE	32.5 ± 3.9*^#^	4.8 ± 0.4	4.2 ± 0.2	3.0 ± 0.2	1.6 ± 0.2	22.6 ± 1.6	15.0 ± 0.4	3.2 ± 0.1	1.4 ± 0.03
*Day 2*	CON	6.7 ± 0.8	2.2 ± 0.2	3.5 ± 0.1	3.6 ± 0.5	1.3 ± 0.1	25.4 ± 2.5	13.4 ± 0.4	2.7 ± 0.2	1.3 ± 0.03
	CE	6.2 ± 0.9	1.8 ± 0.1	3.5 ± 0.2	3.0 ± 0.1	1.4 ± 0.1	25.1 ± 2.5	13.5 ± 0.2	3.0 ± 0.4	1.4 ± 0.1
	HIIE	8.5 ± 1.2	2.7 ± 0.4	4.1 ± 0.3	2.9 ± 0.04	1.4 ± 0.1	25.6 ± 0.9	14.0 ± 0.6	2.4 ± 0.1	1.3 ± 0.1

Values are total content, expressed as μmol of esterified lipid per gram of liver wet weight. *n* = 6 per group. *TAG* Triacylglycerol, *DAG* Diacylglycerol, *ChE* Cholesterol ester, *CL* Cardiolipin, *LyPC* Lysophosphatidylcholine, *PC* Phosphatidylcholine, *PE* Phosphatidylethanolamine, *PS* Phosphatidylserine, *SM* Sphingomyelin. Day 1: 3 h after exercise. Day 2: 28 h following exercise. Statistics by ANOVA. ^^^Main effect of trial, *P* < 0.05. ^&^Trial-by-time interaction, *P* < 0.05. *Significantly different from CON within Day 1, *p* < 0.05. ^#^Significantly different from CE within Day 1, *P* < 0.05

The total content (μmol / gram liver wet weight) for each lipid class is reported in Table 1, as measured by gas chromatography following lipid class fractionation. For hepatic TAG, a significant main effect of trial (*P* < 0.05) and significant trial-by-time interaction (*P* < 0.05) was observed. Post hoc testing indicated that on the day of exercise (Day 1) during post-exercise recovery both CE and HIIE groups exhibit elevated TAG compared to the CON group (*P* < 0.05) and the HIIE group exhibited higher hepatic TAG than the CE group (*P* < 0.05). There were no significant effects of exercise on hepatic TAG content on the day following exercise (Day 2). This intensity-dependent and transient effect of prior exercise on hepatic TAG content in female mice was previously reported [17] using a different TAG analysis method. There were no significant effects of exercise on the total content of other lipid classes (Table 1). However, novel discoveries about correlations between the content of TAG with other lipid classes are discussed below, as well as observations related to acyl chain compositions.

**Fig. 1:**
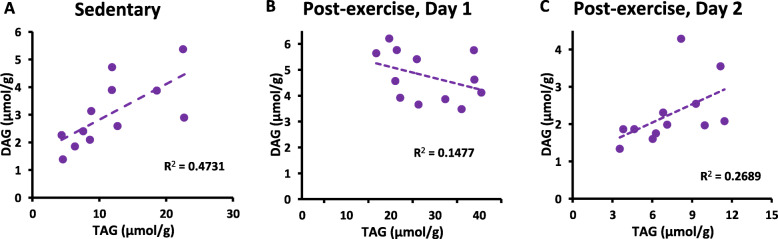
Correlation between total hepatic TAG and total hepatic DAG. **a** CON mice (*P* < 0.05), (**b**) 3 h after exercise (*P* = 0.22, NSD), (**c**) the day following exercise (*P* = 0.08). Day 1: 3 h after exercise. Day 2: 28 h following exercise. CE and HIIE data are pooled in panels **b** and **c**. Analyses by linear regression indicate a statistically significant association between hepatic TAG and DAG under sedentary conditions (CON), a disruption of this association soon after an exercise bout, and a trend (*P* < 0.1) toward resuming the association between TAG and DAG on the day following exercise

**Fig. 2:**
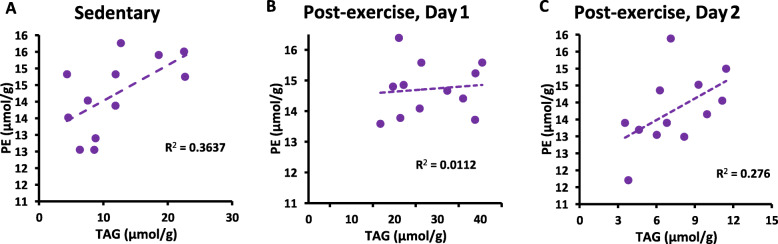
Correlation between total hepatic TAG and total hepatic PE. **a** CON mice (*P* < 0.05), (**b**) 3 h after exercise (NSD, *P* = 0.74), (**c**) the day following exercise (*P =* 0.08). Day 1: 3 h after exercise. Day 2: 28 h following exercise. CE and HIIE data are pooled in panels **b** and **c**. Analyses by linear regression indicate a statistically significant association between hepatic TAG and PE under sedentary conditions (CON), a disruption of this association soon after an exercise bout, and a trend (*P* < 0.1) toward resuming the association between TAG and PE on the day following exercise

To develop a better understanding of the lipidomic context for hepatic TAG content under sedentary conditions and after exercise, linear regression was performed to test association between total content of hepatic TAG and the other lipid classes in the liver. Hepatic TAG content was significantly correlated with hepatic DAG content in CON mouse livers (*P* < 0.05, Fig. 1a). However, this positive association was transiently disrupted following exercise when hepatic TAG was elevated on Day 1, as indicated by analysis of exercised mouse livers (NSD, Fig. 1b). On the day following exercise, there was a trend for the association between hepatic TAG and DAG in exercised mice to correlate with one another (Fig. 1c, *P* < 0.1), suggesting a return to the phenotype of CON mice in exercised mice after a full day to recover. Hepatic TAG was also significantly correlated with hepatic PE in CON mouse livers (*P* < 0.05, Fig. 2a). However, this positive association was transiently disrupted following exercise on Day 1, as indicated by analysis of exercised mouse livers (NSD, Fig. 2b). On the day following exercise, there was a trend for the association between hepatic TAG and PE in exercised mice to correlate with one another (Fig. 2c, *P* < 0.1). This correlation analysis workflow revealed no other significant correlations between hepatic TAG and other lipid classes.

Although the total content of TAG was altered on Day 1 after exercise, there were no significant differences between any groups for the relative abundance of SFA, MUFA, n-3 PUFA, and n-6 PUFA in the hepatic TAG pool; overall, for all liver samples studied, hepatic TAG exhibited the following fatty acid class distribution: SFA, 26.8 ± 0.3%, MUFA. 23.8 ± 0.3%, n-3 PUFA, 11.5 ± 0.2%; n-6 PUFA, 37.9 ± 0.5%. In contrast to TAG, the fatty acid class distribution within hepatic DAG was significantly altered by exercise; ANOVA indicated a main effect of trial for SFA and PUFA (*P* < 0.05) with no significant time-by-trial interactions. Post hoc testing of DAG to explore the main effect of trial indicated that SFA was significantly lower in HIIE than CE and CON, with no difference between CE and CON. Post hoc testing of the main effect of trial also indicated that total PUFA and n-6 PUFA in the DAG pool were significantly higher in HIIE than CE and CON, with no significant difference between CE and CON. These post hoc tests specifically explored effects of exercise collectively for Day-1 (3 h after exercise) and Day-2 (28 h after exercise). The relative content of n-6 PUFA in DAG on Day 1 (Con, 42.2 ± 1.6%; CE, 43.8 ± 1.6%; HIIE, 45.8 ± 1.2%) and Day 2 (Con, 38.4. ± 1.2%; CE, 39.0 ± 0.5%; HIIE, 42.6 ± 0.7) indicated a modest but sustained effect of a single bout of HIIE on DAG acyl chain composition. The relative content of SFA in DAG on Day 1 (Con, 22.3 ± 0.8%; CE, 21.6 ± 0.7%; HIIE, 20.9 ± 0.6) and Day 2 (Con, 25.5. ± 1.1%; CE, 26.8 ± 0.8%; HIIE, 23.6 ± 0.6) indicated changes that were in the opposite direction of the changes in n-6 PUFA after HIIE. No other lipid classes showed a change in the relative abundance of total SFA, MUFA, or PUFA after exercise; however, as discussed below, further exploration of the molecular signature of lipid classes after exercise was carried out by PCA, based upon acyl chain composition.

**Fig. 3:**
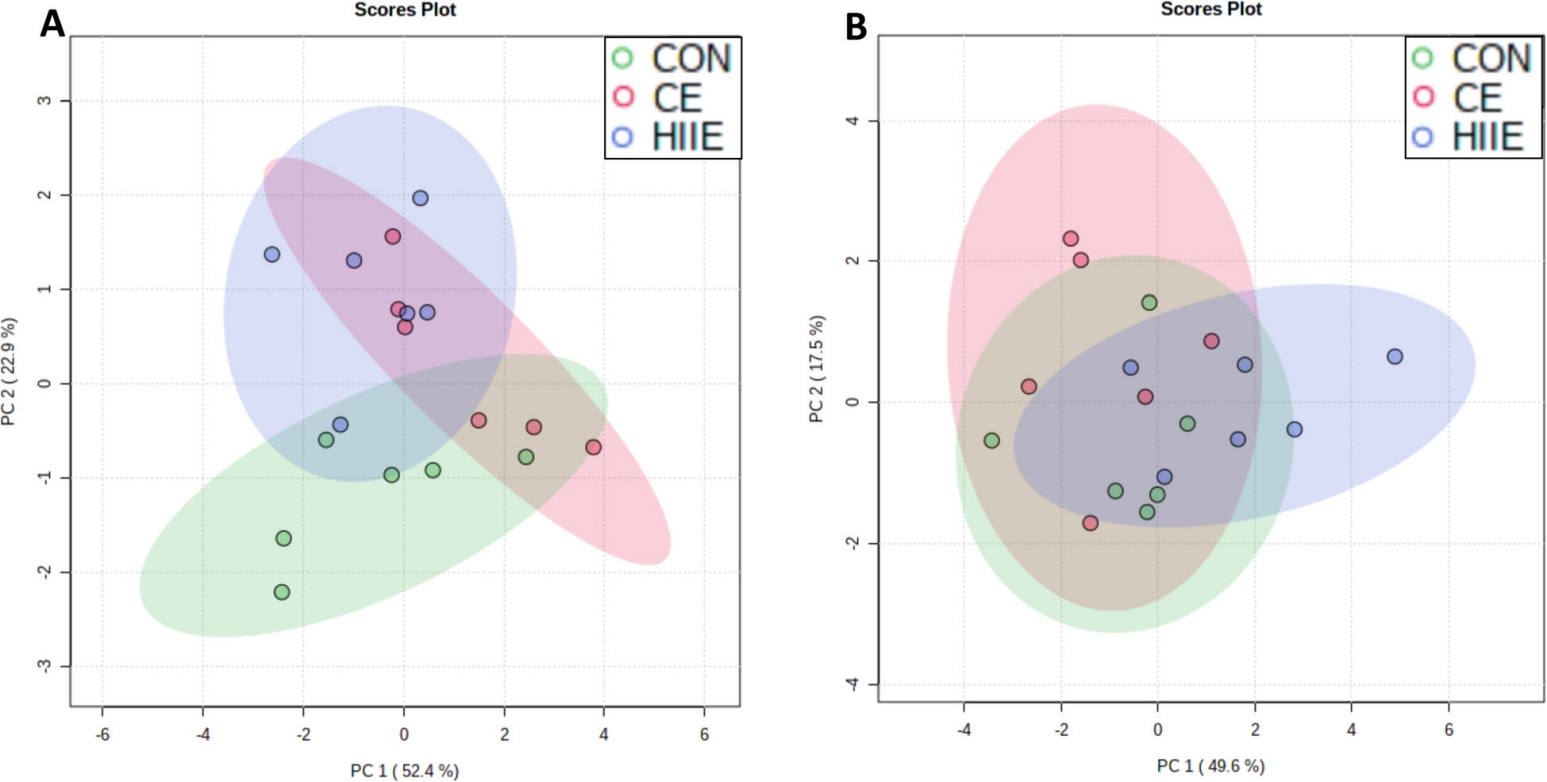
Principal Component Analysis of Hepatic Triacylglycerol. **a** The day of exercise (Day 1, 3 h after exercise), (**b**) the day after exercise (Day 2, 28 h after exercise). The relative fatty acid composition of triacylglycerol (TAG) was analyzed by PCA to assess remodelling of the acyl chain composition as a means of identifying the group assignment of each mouse liver sample. CON, green; CE, red; HIIE, blue

**Fig. 4:**
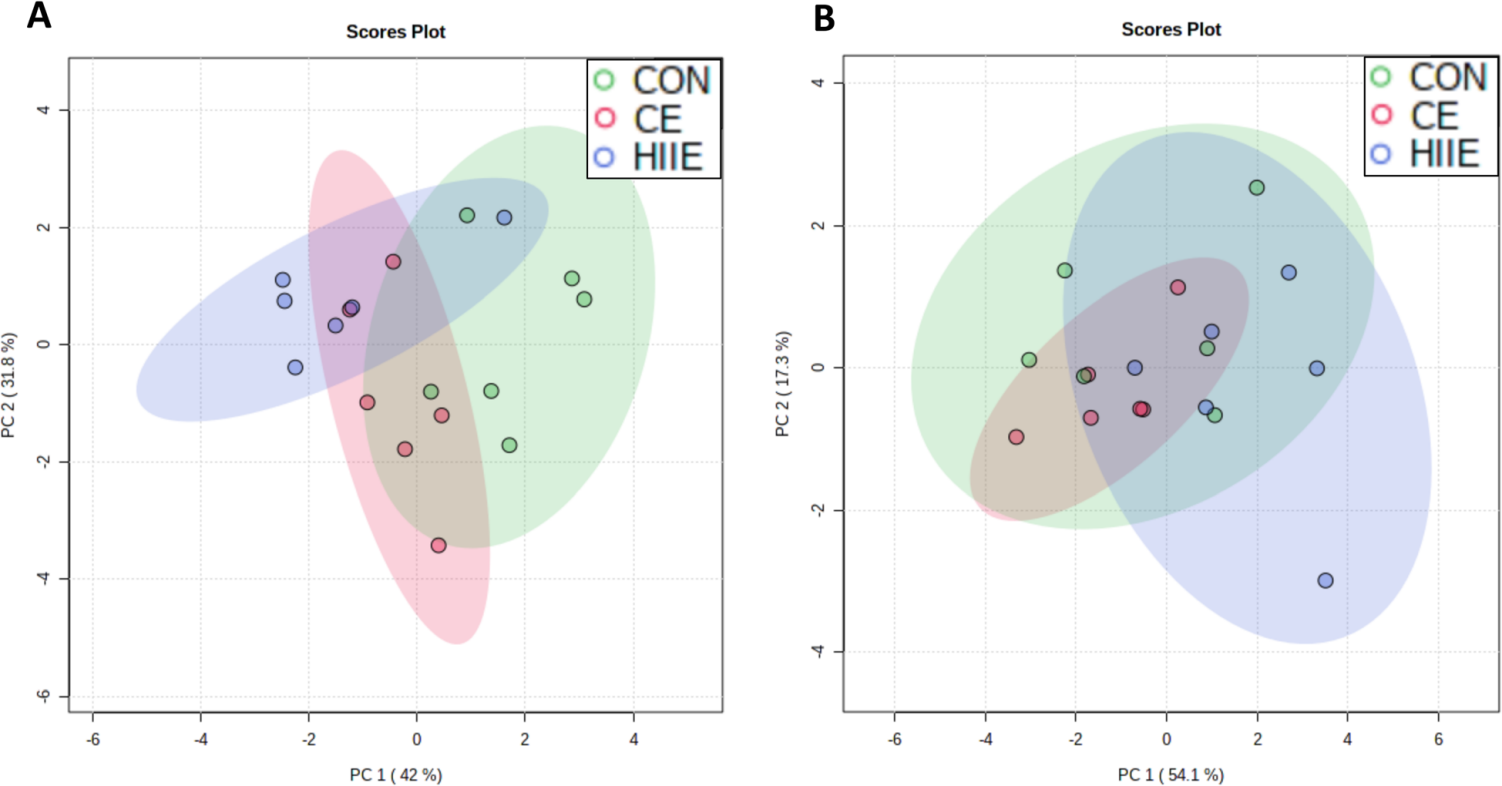
Principal Component Analysis of Hepatic Diacylglycerol. **a** The day of exercise (Day 1, 3 h after exercise), (**b**) the day after exercise (Day 2, 28 h after exercise). The relative fatty acid composition of diacylglycerol (DAG) was analyzed by PCA to assess remodelling of the acyl chain composition as a means of identifying the group assignment of each mouse liver sample. CON, green; CE, red; HIIE, blue

**Fig. 5:**
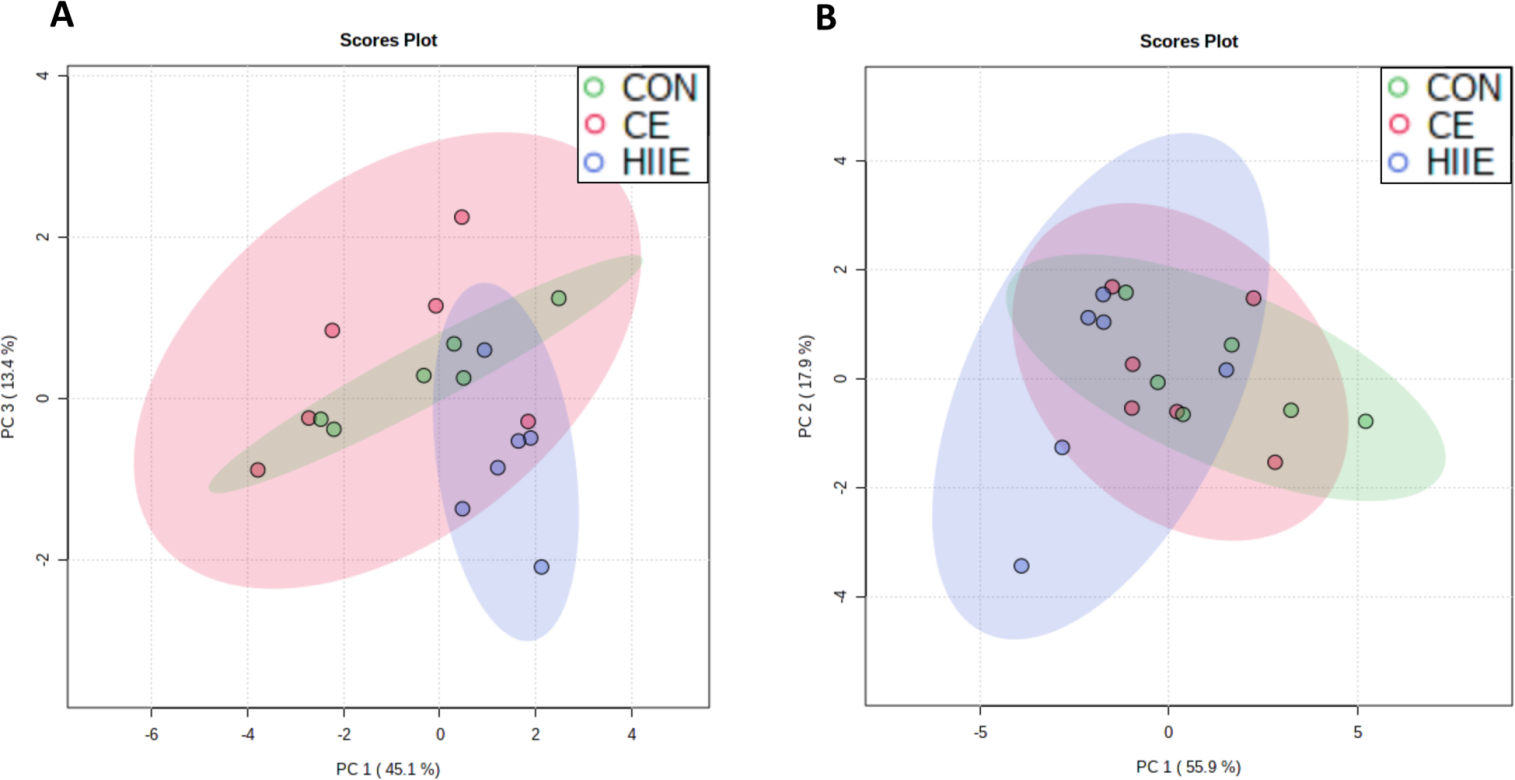
Principal Component Analysis of Hepatic Phosphatidylethanolamine. **a** The day of exercise (Day 1, 3 h after exercise), (**b**) the day after exercise (Day 2, 28 h after exercise). The relative fatty acid composition of phosphatidylethanolamine (PE) was analyzed by PCA to assess remodelling of the acyl chain composition as a means of identifying the group assignment of each mouse liver sample. CON, green; CE, red; HIIE, blue

As the main findings presented above are related to TAG, DAG, and PE, here PCA plots are presented for the relative fatty acid composition of these lipid classes. For TAG, Fig. 3a indicates that CON and HIIE achieved nearly complete separation on Day 1, while the lower-intensity exercise group (CE) overlaid with both CON and HIIE. This observation is consistent with the concept of intensity-dependent responses of lipid metabolism. Day 2 showed no meaningful group separations for TAG (Fig. 3b). For PCA of TAG, PC1 accounted for 52.4% of the data variance on Day-1 and 49.6% on Day-2; PC2 accounted for 22.9% of data variance on Day-1 and 17.5% on Day-2. As stated in Methods, fatty acids with a VIP score above 1 were included in the PCA models for TAG (Day-1: 16:1n-7, 18:1n-9, 18:3n-6, 20:1n-9, 20:2n-6, 22:5n-3; Day-2: 16:0, 18:1n-7, 18:2n-6, 18:3n-6, 20:2n-6, 22:4n-6, 22:5n-3, 22:5n-6). Figure 4a indicates that CON and HIIE achieved nearly complete separation for the DAG lipid class on Day 1, with the exception of a single mouse in the HIIE group that appeared to be an outlier, while the CE group appearing to overlay with both CON and HIIE. Day 2 showed no meaningful trends toward group separation for DAG (Fig. 4b). For PCA of DAG, PC1 accounted for 42.0% of the data variance on Day-1 and 54.1% on Day-2; PC2 accounted for 31.8% of data variance on Day-1 and 17.3% on Day-2. Fatty acids with a VIP score above 1 were included in the PCA models for DAG (Day-1: 16:0. 16:1n-7, 18:3n-6, 18:4n-3, 20-1n-9, 22:5n-3, 22:5n-6; Day-2: 16:0, 18:0, 18:1n-7, 18:2n-6, 18:3n-6, 18:4n-3, 20:3n-6, 20:4n-6). Figure 5a indicates that CON and HIIE achieved nearly complete separation on Day 1 for the PE lipid class, with the exception of a single mouse in the HIIE group that appeared to be an outlier, while the CE group overlaid with both CON and HIIE. Day 2 showed no meaningful trends toward group separation for PE (Fig. 5b); testing additional combinations of principal components (e.g., PC1 with PC2 or with PC3) did not lead to any meaningful group separation on day 2. For PCA of PE, PC1 accounted for 45.1% of the data variance on Day-1 and 55.9% on Day-2; PC3 accounted for 13.4% of data variance on Day-1, and PC2 accounted for 17.9% on Day-2. Fatty acids with a VIP score above 1 were included in the PCA models for PE (Day-1: 16:0, 16:1n-7, 18:2n-6, 20:3n-9, 20:4n-6, 20:5n-3, dimethoxy acetal 16:0, dimethoxy acetal 18:0; Day-2: 16:1n-7, 18:0, 18:1n-7, 18:3n-3, 20:1n-9, 20:3n-9, 20:5n-3, 22:4n-6, dimethoxy acetal 16:0, dimethoxy acetal 18:0). Review of PCA results in MetaboAnalyst indicated that it was the same HIIE mouse that appeared to be an outlier on Day 1 for TAG, DAG, and PE; however, all data points are shown in the PCA to display the full variability observed.

## Discussion

The liver transiently accumulates TAG following a single exercise bout, particularly if the exercise bout is challenging. This ability to rapidly expand the hepatic TAG pool size, referred to here as lipogenic flexibility, has been discovered previously but remains poorly understood. The present report describes observations of lipogenic flexibility, based upon findings from lipidomics analysis of the liver following a moderate-intensity exercise bout (CE) and a high-intensity exercise bout (HIIE) in mice. Through the present work, the presence of transient liver TAG accumulation after exercise has been rigorously confirmed, and the findings have been elaborated through the analysis of various lipid classes in the liver. Associations between liver TAG content and other lipids in CON mice were discovered, and these associations were transiently altered by a single exercise bout; the implications of these findings are discussed below. Discussion below also addresses intensity-dependence of the lipogenic flexibility response, as indicated by robust responses particularly to HIIE for TAG content changes as well as additional metabolic impacts indicated by PCA plots of acyl chain compositions. Finally, the overall implications of the present results are discussed in relation to the mammalian exercise response and the implications for metabolic health.

The exercise-responsive lipogenic flexibility in the liver is depicted primarily by a significant accumulation of hepatic TAG after exercise, which returns back toward baseline CON levels by the following day. Accurate measurement of TAG is truly essential for work aimed at characterizing this phenomenon. The investigative team authoring this report have observed this hepatic TAG phenomenon by two independent analytical procedures, including a colorimetric biochemical TAG assay published previously [17] and an analytical chemistry-based approach using thin layer chromatography followed by gas chromatography as reported in the present report (Table 1). Both approaches led to the observation of an intensity-dependent accumulation of TAG in the liver of female mice after exercise which resolved by the following day. In the biochemical work published previously, both sexes were studied, while for the current lipidomics approach a single sex was selected for sample submission to a lipidomics analysis laboratory. In these female mouse liver samples, there was a high degree of correlation between TAG results from the two methods, with a slope near unity; thus, one can be confident that previous observations are robust and confirmed by the present TAG analysis. What’s more, in the present report a broad range of lipids are reported, leading to an elaboration of the lipogenic flexibility phenomenon beyond the measurement of TAG and its lipid droplet-related proteins that were reported previously [17].

The present results in Fig. 1a indicate that under sedentary conditions, there is a significant positive correlation between hepatic TAG and DAG content. This finding is consistent with previous knowledge that TAG accumulation in the liver is associated with DAG accumulation [1–5], which can then lead to impairments in metabolic health [2, 7, 8]. However, when transient TAG accumulation is triggered in the liver by exercise, the correlation between TAG and DAG is disrupted (Fig. 1b); this indicates that the metabolically inert TAG pool can accumulate in this scenario without being strongly associated with lipotoxic DAG accumulation. As the hepatic TAG content returned back toward CON levels during recovery on the day after exercise, its association with DAG content was also nearly reestablished. Transient enhancement of perilipin-2 (pln2) expression in the liver after exercise may stabilize lipid droplets as the TAG pool size expands [17]. The previous results for pln2 expression [17] indicate enhanced capacity after exercise to transiently sequester TAG within hepatic lipid droplets, while DAG residing outside lipid droplets would not be sequestered by pln2.

Under sedentary conditions (CON), hepatic TAG is also significantly correlated with hepatic PE (Fig. 2a), while this statistical association is disrupted during the rapid expansion of the hepatic TAG pool following a single exercise bout (Fig. 2b). This is followed by a trend toward the correlation between TAG and PE being reestablished in exercised mice on the day after the exercise bout (Fig. 2c). The positive association between TAG and PE in sedentary mice (CON) or exercise-recovered mice could be potentially expected, because cellular PE content could affect lipid droplet biology. PE is a component of the phospholipid monolayer surrounding lipid droplets [28] and may be involved in formation [29] or fusion of lipid droplets [30]. Thus, a relationship between TAG accumulation and tissue PE content seems understandable. However, during the transient TAG accumulation in the liver that occurs rapidly in response to exercise, the TAG pool expansion outpaces any PE biosynthesis.

When considering the variety of lipid classes analyzed in this study, it is clear that TAG was the most responsive to exercise. This indicates that the lipidomic response to exercise in the liver may be primarily related to fuel metabolism rather than changes in structural lipids within the cell. While TAG was the only lipid class showing a change in concentration, DAG was the lipid class that showed a change in the distribution of fatty acid classes within the esterified lipid pool, with exercise-induced increases in total PUFA and n-6 PUFA alongside the corresponding reduction in SFA content. While these statistically significant changes in DAG composition may be reasonably modest in magnitude, it is noteworthy that they occurred in response to only a single bout of exercise, and they were sustained even the day following exercise. The mice in the study reported here consumed the 5 K52 diet which contains a reasonably substantial n-6 PUFA abundance (46% of fatty acids) [25]. After HIIE the PUFA content in liver DAG rose to approach this value of PUFA expected from the diet, which may be caused by an exercise-induced release of dietary fatty acids that were stored in adipose tissue. Furthermore, while discussing the impacts of exercise on hepatic lipids, it should be noted that the most substantial impacts were following HIIE, which is an exercise approach that exhibits particularly potent impacts upon health and metabolism [17, 26, 31–36]. The remodeling of PUFA content in DAG occurred after HIIE but not CE, and the response of TAG concentration was enhanced following HIIE in comparison to CE. Furthermore, principal component analysis indicated that the separation between HIIE and CON to be more notable than separation between CE and CON, suggesting potentially a more potent impact upon turnover of cellular lipids in the liver. While PCA plots, even with an individual mouse as an outlier, indicated a general separation between HIIE and CON for the lipid classes reported (TAG, DAG, PE), the CE data points were broadly dispersed and overlapped with the CON group. As a whole, the data are supportive of a biologically distinct impact of HIIE in comparison to CE, even when these exercise types are matched for distance, duration, and energy expenditure. Thus, it appears that intermittently challenging exercise is more metabolically potent in the liver than sustained mild exertion.

In order to understand the metabolic events that lead to exercise-induced changes in hepatic lipids, the timing of the changes in TAG accumulation could be considered. In this work liver tissue was collected 3 h after exercise and the following day. The accumulation of hepatic TAG seen at 3 h after exercise hypothetically could have occurred during exercise, during the first few hours of post-exercise recovery, or during both time periods. There have been some reports indicating that TAG has already accumulated in the liver at the end of the exercise bout in humans [11, 13, 15], mice [37], and rats [24]. In another study on human subjects, TAG accumulation did not occur during exercise but subsequently accumulated during four h of recovery [15]; a similar observation was made studying mice, in which hepatic TAG did not accumulate during exercise but subsequent accumulated during three h of recovery [38]. In contrast, in a study on rats hepatic TAG accumulated during exercise but began to recover soon after, substantially returning toward baseline even within an hour of recover [9]. Alternatively, in mice the hepatic TAG that accumulated during exercise was fully maintained 3 h after exercise [37]. Overall, it appears that TAG can potentially accumulate in the liver both during exercise and during hours following exercise, with a sustained elevation typically lasting for hours, but with recovery time ranging from 1 h to perhaps approximately 24 h. Nutritional status likely plays a role, and investigation of the effects of food/beverage intake after exercise deserves attention in the future. If accumulation of TAG in the liver is driven by plasma FFA concentration, then accumulation could be promoted both during and after exercise; exercise indeed leads to increased plasma FFA turnover and concentration both during exercise and during hours of post-exercise recovery [20]. As discussed in the Introduction, control of lipolysis during and after exercise may have evolved based upon the fuel supply needs of skeletal muscle. However, as enhanced lipolysis drives an elevated FFA concentration in plasma and thus increased FFA uptake down concentration gradients into working muscle [39–41], this response places a metabolic burden upon non-contracting muscle and other organs such as the liver that will be presented with circulating FFA levels that are beyond their needs. It appears that enhanced circulating FFA, though useful for fuel trafficking from storage sites to sites of use, can place a burden and enhanced lipotoxicity risk upon peripheral tissues. Ideally, for preservation of metabolic health, this elevated FFA would be buffered into the TAG pool intracellularly, which is metabolically inert, rather than being stored in lipotoxic intermediate pools such as DAG.

While it appears plausible that lipogenic flexibility in the liver after exercise is driven largely by uptake of plasma FFA down concentration gradients, it is worth noting that the liver also possesses an elaborate network of enzymatic control of lipid metabolism. For example, diet can exhibit potent effects upon the lipogenic pathway through sterol regulatory element-binding protein 1c SREPB-1c [42, 43]. This pathway leads to expression changes of lipogenic enzymes such as fatty acid synthase, stearoyl CoA desaturase, and acetyl CoA carboxylate [42, 43]. Also diet can chronically modulate expression and activity of peroxisome proliferator activated receptor-α (PPAR-α) which impacts expression of enzymes related to the fat oxidation pathway [42–44] and mitochondrial function [44, 45]. Chronic exercise training can improve mitochondrial function in the liver through effects on sirtuin expression, regulation of enzyme activities and substrate selection, or through mitochondrial biogenesis [46]. Chronic exercise training may limit steatosis through actions upon these lipogenic and oxidative pathways, but it is expected that remodeling of mitochondrial metabolism would require many exercise bouts over a significant time period. The acute effect of each exercise bout would likely act through other means that are distinct from these impacts of diet and chronic exercise. Despite presence of a control network acting chronically through modulation of transcription factors, substrate supply also remains an important controller of tissue TAG synthesis and accumulation. When plasma FFA concentration is elevated, as occurs during and after exercise, hepatic TAG accumulation is expected because of enhanced FFA uptake. Furthermore, the enhanced pln2 expression in the liver after exercise [17] may impair access of lipolytic enzymes to the hepatic lipid droplets, promoting transient TAG accumulation. To summarize, exercise may very rapidly alter intracellular TAG content through pathways that do not necessarily require changes in lipogenic or fat oxidation enzyme expression levels.

It is understood that the liver after exercise is able to exhibit a lipogenic flexibility, supported by pln2 expression, that allows rapid expansion of the TAG pool and buffering of FFA into this inert pool [17]. Next, it would be useful to consider this phenomenon exhibited by the liver in the context of lipid changes occurring in other tissues in response to exercise and related stressors. During exercise, the amount of intramuscular TAG declines in the exercised muscles, while TAG tends to accumulate elsewhere. TAG accumulates in the liver (present results) and even in skeletal muscle that was not actively recruited for the exercise bout [12]. During fasting, which also stimulates lipolysis but is not associated with vigorous muscle contraction, skeletal muscle actually accumulates TAG as seen in laboratory animals by biochemical analysis [47] and in human subjects by measuring intramyocellular lipid by MRS [48–52]. As with exercise, fasting leads to accumulation of TAG in the liver as observed in rodent studies [53–56] and accumulation of intrahepatocellular lipid (presumably mostly TAG) as observed by non-invasive MRS in human subjects research [57]. It is important to keep in mind that the acute response to each bout of a stressor is not necessarily qualitatively similar to the chronic stress response. Specifically, chronic caloric restriction typically reduces hepatic TAG concentration [58] while acute fasting leads to elevation of hepatic TAG [57]. Similarly, in some cases chronic exercise training modestly reduces hepatic TAG [6, 19]; however, each acute bout of exercise transiently raises hepatic TAG [11, 13–16], even when pre-exercise hepatic TAG is high as in patients with non-alcoholic fatty liver disease (NAFLD) [14]. Specifically, the exercise modalities reported here acutely raise hepatic TAG on the day of exercise, but in mice that were chronically trained by CE or HIIE, with liver tissue collection on the day following the last exercise bout, hepatic TAG content in exercised mice was not elevated above CON [26]. While chronic adaptations are certainly meaningful, the ability to buffer excess plasma FFA into the hepatic TAG pool is likely to be metabolically critical when a stressor is acutely applied that increases circulating FFA.

### Study strengths and limitations

The present study was designed to elucidate effects of acute exercise bouts upon subsequent resting metabolism. This investigation into the post-exercise recovery period benefited from inclusion of two time points. Three h after exercise depicts the early recovery phase (Day 1) while the time point on the following day (Day 2) represents the late recovery phase as metabolism returned back toward baseline. It is a strength that a time-matched CON group was included, as it is critical to control for diurnal variation and changes in energy balance across the day. It is also a strength of the study that two different exercise types were tested, such that the findings could have implications for multiple exercise prescription options. It may be advantageous in future investigations to include earlier time points as well (such as collecting tissues immediately after exercise) in order to add additional temporal resolution to the findings. Secondly, while in this work non-obese female mice were studied, it would be useful to study both sexes as well as obesity when further exploring the topic of lipogenic flexibility in the future.

## Conclusions

In summary, the hepatic lipidome responds to exercise in an intensity-dependent manner. Furthermore, while considering CE and HIIE in the same regression analysis, the relationship between TAG content and that of other lipid classes was discovered to be altered when hepatic TAG content fluctuates after exercise. It may be useful in the future to further explore the implications of exercise-induced lipogenic flexibility in the liver. For example, it could be tested if there are populations that are unable to rapidly expand tissue TAG storage at ectopic deposition sites when plasma FFA rise. Another future direction could be to test whether preventing the exercise-induced fluctuations in hepatic TAG content would alter the health impacts of exercise. As the exercise-induced transient changes in the hepatic lipidome become well-appreciated and characterized by the scientific community, it will become critical to explore the implications for human health. While clinically it is desirable to minimize hepatic TAG storage in obese individuals, as excessive accumulation can lead to insulin resistance and NAFLD, this general goal must be considered within the context of the normal fluctuations of hepatic TAG content. Exercise is recommended to improve health, and exercise can induce rapid fluctuations in hepatic TAG content over the course of the day. Exercise is well-accepted as a clinically useful treatment for various maladies, yet an acute exercise bout transiently raises hepatic TAG. The scientific community may need to reconsider how hepatic lipid content is monitored. Rather than solely considering hepatic lipid content in the overnight fasted state as a health indicator, the daily fluctuations may be even equally important in determination of health and disease risk.

## Data Availability

The datasets used and/or analyzed during the current study are available from the corresponding author on reasonable request.

## Abbreviations

ANOVA: Analysis of variance
CL: Cardiolipin
ChE: Cholesterol ester
CE: Continuous exercise
CON: Control
DAG: Diacylglycerol
FFA: Free fatty acid
HIIE: High-intensity interval exercise
LyPC: Lysophosphatidylcholine
MRS: Magnetic resonance spectroscopy
MUFA: Monounsaturated fatty acid
NAFLD: Non-alcoholic fatty liver disease
PC: Phosphatidylcholine
PE: Phosphatidylethanolamine
PS: Phosphatidylserine
PPAR-α: Peroxisome proliferator activated receptor-α
PUFA: Polyunsaturated fatty acid
PCA: Principal component analysis
SM: Sphingomyelin
SFA: Saturated fatty acid
SREBP-1C: Sterol regulatory element-binding protein 1c
TAG: Triacylglycerol
VIP: Variable importance in the projection
